# Dual Antimicrobial and Antiproliferative Activity of TcPaSK Peptide Derived from a *Tribolium castaneum* Insect Defensin

**DOI:** 10.3390/microorganisms9020222

**Published:** 2021-01-22

**Authors:** Aida Robles-Fort, Inmaculada García-Robles, Wasundara Fernando, David W. Hoskin, Carolina Rausell, María Dolores Real

**Affiliations:** 1 Department of Genetics, University of Valencia, Burjassot, 46100 Valencia, Spain; aida.robles@uv.es (A.R.-F.); garciai@uv.es (I.G.-R.); carolina.rausell@uv.es (C.R.); 2 Department of Pathology, Faculty of Medicine, Dalhousie University, Halifax, NS B3H 4R2, Canada; wasufer@Dal.Ca (W.F.); d.w.hoskin@dal.ca (D.W.H.); 3 Department of Microbiology and Immunology, Faculty of Medicine, Dalhousie University, Halifax, NS B3H 4R2, Canada; 4 Department of Surgery, Faculty of Medicine, Dalhousie University, Halifax, NS B3H 4R2, Canada

**Keywords:** antimicrobial peptides, insect defensins, *Staphyloccoccus aureus*, triple negative breast cancer, SWATH

## Abstract

Antimicrobial peptides (AMPs) found in the innate immune system of a wide range of organisms might prove useful to fight infections, due to the reported slower development of resistance to AMPs. Increasing the cationicity and keeping moderate hydrophobicity of the AMPs have been described to improve antimicrobial activity. We previously found a peptide derived from the *Tribolium castaneum* insect defensin 3, exhibiting antrimicrobial activity against several human pathogens. Here, we analyzed the effect against *Staphyloccocus aureus* of an extended peptide (TcPaSK) containing two additional amino acids, lysine and asparagine, flanking the former peptide fragment in the original insect defensin 3 protein. TcPaSK peptide displayed higher antimicrobial activity against *S. aureus*, and additionally showed antiproliferative activity against the MDA-MB-231 triple negative breast cancer cell line. A SWATH proteomic analysis revealed the downregulation of proteins involved in cell growth and tumor progression upon TcPaSK cell treatment. The dual role of TcPaSK peptide as antimicrobial and antiproliferative agent makes it a versatile molecule that warrants exploration for its use in novel therapeutic developments as an alternative approach to overcome bacterial antibiotic resistance and to increase the efficacy of conventional cancer treatments.

## 1. Introduction

Increasing resistance to conventional antibiotics represents one of the most critical menaces to human health and has fostered the development of antimicrobial peptides (AMPs) as next-generation drugs to selectively combat pathogens. AMPs have several advantages over conventional antibiotics such as slower emergence of resistance, broad-spectrum antibiofilm action and host immunomodulatory activities [1,2]. 

AMPs are key components of innate immunity, providing the first line of defense of eukaryotic organisms against diverse microbial infections [3]. Accordingly, insects, which are the largest group of living organisms [4] and rely solely on innate immune reactions for protection against microorganisms [5], produce a larger repertoire of AMPs than any other taxonomic group [6]. At present, several structural families of AMPs from insects have been described, and novel AMPs are continuously being discovered [7]. Due to their potent antibacterial, antifungal and antiviral activities, insect peptides with antimicrobial activity have recently attracted increased attention as potential therapeutic agents for medical applications such as treatment of multidrug resistant bacterial infections [7]. 

Insect AMPs are mainly cationic, and in their folded state, they are also amphipathic, with hydrophilic and hydrophobic regions mediating their solubility in phospholipid cell membranes. These properties facilitate their binding to bacterial membranes and their membrane disruptive activity, which is thought to be the primary microbial killing mechanism. However, interactions with diverse intracellular targets have also been reported to contribute to the efficacy of some insect AMPs against pathogens [8]. 

Based on their amino acid sequence and structures, most insect AMPs fall into three categories: (i) Linear α-helical AMPs, such as cecropins, moricin, sarcotoxin, and melittin, which are present in a wide range of insect orders, including coleopterans, dipterans, and lepidopterans; (ii) Linear proline or glycine-rich AMPs that include drosocin, apidaecin, formaecin, and pyrrhocoricin; and (iii) Cysteine-stabilized AMPs that are common in most insects and include defensins or defensin-like compounds, such as gallerimycin, heliomycin, sapecins, drosomycin, spodoptericin, and phormicins [9,10]. 

Insect defensins have been reported to possess broad-spectrum activity against bacteria and fungi and they have been proposed as promising candidates for the development of novel antibiotic lead molecules, due to their structural stability and amenability to peptide engineering [11]. Defensins from insects share with other organisms’ defensin peptides a particular structural topology known as cysteine-stabilized αβ motif (CSαβ), that confers on them high stability against heat and proteases. Insect defensins contain six conserved cysteines that form a typical arrangement of three disulfide bonds. Two disulfide bonds connect the C-terminal β-sheet and the α-helix and the third connects the N-terminal loop with the second β-sheet. Interestingly, although all insect defensins have in common this structural motif, their primary sequence, as well as their spectrum of antimicrobial activity, varies considerably. Therefore, structure activity studies are currently being promoted to gain more insight into the molecular mechanisms underlying the bioactivity of insect defensins [11]. 

As for other organisms’ AMPs, which also exhibit anticancer activities and are termed anticancer peptides (ACPs) [12,13], insect defensins have been reported to possess antiproliferative activity. In cancer cells, the increased abundance of negatively charged phosphatidylserine (PS) on the outer leaflet, the overexpression of other negatively charged molecules such as heparan sulfate and the increased transmembrane potential and membrane fluidity result in enhanced interactions with some AMPs [14]. Several studies have explored the anticancer effect of insect AMPs [15], and among them, insect defensins. Iwasaki et al. [16] designed and synthesized four enantiomeric 9-mer peptides on the basis of 43-mer insect defensins from two beetles and despite all of them maintaining similar bacterial membrane disruptive activity as the original peptides, they differentially inhibited the growth of several cancer cell lines and had no effect against normal leukocytes. A strong correlation was observed between negatively charged PS density in the cancer cell membrane surface and sensitivity to D-9-mer peptides in various cancer cell lines. The group of Hwang reported the anticancer activity of a synthetic 9-mer dimer CopA3 peptide based on the defensin-like peptide Coprisin, isolated from the bacteria-immunized dung beetle *Copris tripartitus* [17], as well as the synthetic homodimer peptide analogue HaA4 derived from harmoniasin, a defensin-like antimicrobial peptide identified from the ladybug *Harmonia axyridis* [18]. These peptides were shown to have therapeutic potential to treat pancreatic and hepatocellular cancers, and leukemia, respectively.

In our previous work, while studying the immune response of the coleopteran insect *Tribolium castaneum* following *Bacillus thuringiensis* intoxication, we identified a peptide fragment of this insect’s defensin 3 (Tcdef3) with antimicrobial activity against *Escherichia coli*, *Staphylococcus aureus*, and *Candida albicans* strains [19]. Now, we have analyzed the antimicrobial and antiproliferative activity of a peptide fragment (named TcPaSK), containing the Tcdef3 sequence and two additional amino acids present in the original *T. castaneum* insect’s defensin 3 primary sequence, lysine at the N-terminus and asparagine at the C-terminus. As proposed for other AMPs [14], these amino acids possess physicochemical properties that may improve the peptide antimicrobial activity.

## 2. Materials and Methods

### 2.1. Peptide Synthesis

Peptides Tcdef3 and TcPaSK were synthesized and obtained at a purity grade of 90% and 95.8%, respectively, by HPLC (GenScript Co., Piscataway, NJ, USA). The human defensin hBD-3 was synthesized and obtained at a purity grade of >99% by HPLC (PeptaNova GmbH, Sandhausen, Germany). The peptide sequences were: Tcdef3:

VNHAACAAHCLLKRKRGGYCNKRRICVCR;
TcPaSK: KVNHAACAAHCLLKRKRGGYCNKRRICVCRN;
hBD-3: GIINTLQKYYCRVRGGRCAVLSCLPKEEQIGKCSTRGRKCCRRKK

### 2.2. Reagents and Chemicals

Dulbecco’s modified Eagle’s medium (DMEM), fetal bovine serum (FBS), N-2-hydroxyethylpiperazine-N′-2-ethanesulfonic acid (HEPES), L-glutamine, penicillin–streptomycin (Pen-strep), fungizone, trypsin [0.25% with EDTA (1×)], TryPLE Express, propidium iodide (PI) and Oregon Green 488 were purchased from Life Technologies Inc. (Burlington, ON, Canada). 5-[3-(carboxymethoxy) phenyl]-3-(4,5-dimethyl-2-thiazolyl)-2-(4-sulfophenyl)-2H-tetrazolium inner salt (MTS) assay kit was purchased from Promega (Madison, WI). Paraformaldehyde was purchased from Bioshop Canada Inc. (Burlington, ON, Canada).

### 2.3. Cell Culture Conditions

The bacterial strain used was *Staphylococcus aureus* subsp. *aureus* CECT 4013. Cells were grown in LB medium (peptone 1%, yeast extract 0.5% and NaCl 1%) at 37 °C.

For cell viability assays and cell proliferation assays, MDA-MB-231 human triple negative cancer (TNBC) cells and 4T1 mouse mammary carcinoma cells were obtained from Dr. S. Drover (Memorial University of Newfoundland, St. John’s, NL, Canada) and Dr. D. Waisman (Dalhousie University, Halifax, NS, Canada), respectively. Cells were grown in DMEM supplemented with 10% heat-inactivated FBS, 100 U/mL penicillin, 100 μg/mL streptomycin, 2mM L-glutamine and 5mM HEPES (pH 7.4). The cells were maintained at 37 °C in an atmosphere of 10% CO_2_. HC11 mouse mammary epithelial cells were kindly provided by Dr. H.S. Ro (Dalhousie University, Halifax, NS, Canada). Cells were grown in DMEM supplemented with 10% heat-inactivated FBS, 100 U/mL penicillin and 100 μg/mL streptomycin. At 90% confluence, cells were passaged by detachment with trypsin-EDTA, followed by centrifugation at 500× *g* for 5 min and then resuspension in fresh medium.

For cell cycle and proteomic analyses, MDA-MB-231 human TNBC cells were purchased from the American Type Culture Collection (ATCC; Manassas, VA, USA). Cells were grown in DMEM supplemented with 10% FBS, 1% penicillin, 1% streptomycin and 0.1% fungizone. The cells were maintained at 37 °C in a humidified atmosphere of 5% CO_2_.

### 2.4. Minimal Inhibitory Concentration (MIC)

TcPaSK peptide MIC against *Staphylococcus aureus* (CECT 4013) was determined by the broth microdilution method [20], according to EUCAST and CLSI guidelines. In brief, overnight culture was diluted in LB broth to obtain a bacterial suspension of 1 × 10^8^ cfu mL^−1^ that was further diluted 1:100 and added to wells containing medium without peptide (grow control) or containing serial dilutions of two-fold concentrated peptide. Final concentration of bacterial inoculum was 5 × 10^5^ cfu mL^−1^, and final concentration of peptide ranged from 128 to 0.25 µg/mL. The 96-well plate was incubated at 37 °C for 16 to 20 h. The MIC was determined as the lowest concentration, showing no visible growth compared to the control without peptide. 

### 2.5. Hemolytic Activity

TcPaSK peptide hemolytic activity was determined following [21]. Human 0 negative whole blood sample was obtained from a single voluntary donor, collected in tubes containing EDTA as anticoagulant and stored at 4 °C before use. Blood was washed twice with PBS, centrifuged for 8 min at 700× *g* and resuspended at 10% (*v/v*) in PBS. For the last washing, 4 mL of PBS were added, the mixture was centrifuged at 1000× *g* for 8 min and the supernatant was discarded. Then, 40 µL of red blood cells (RBC) were diluted in 8 mL PBS to yield a 0.5% *v/v* RBC suspension.

The assay was performed in a V-bottom 96-well polypropylene deep well plate (Axygen, Corning, NY, USA). The peptide was tested in serial dilutions in PBS, with final concentrations ranging from 150 to 2.35 µM. As positive control, 1.25 µM of melittin (Sigma-Aldrich, Saint Louis, MO, USA), considered the “gold-standard positive control” [21], was used, and PBS as negative control. Each well was mixed with equal volume of 0.5% *v/v* RBC suspension. The plate was sealed with Biorad Microseal film, and incubated for one hour at 37 °C. Finally, it was centrifuged at 1000× *g* for 10 min at room temperature. Sixty µL of supernatants were transferred to a clear flat-bottom 96-well polystyrene microplate (Fisher Scientific, Hampton, NH, USA). The absorbance was measured at λ = 414 nm using a plate reader (Tecan, Männendorf, Switzerland). The percentage of hemolysis was calculated with the following equation:

$$\%Hemolysis=\;\frac{\mathrm{Abs}\left(\mathrm{sample}\right)-\;\mathrm{Abs}\left(\mathrm{NegAvg}\right)}{\mathrm{Abs}\left(\mathrm{PosAvg}\right)-\;\mathrm{Abs}\left(\mathrm{NegAvg}\right)}\times 100$$

### 2.6. Antimicrobial Activity

*S. aureus* cells were grown overnight in LB liquid medium at 37 °C in agitation. Aliquots in fresh liquid medium were allowed to grow until an optical density at 600 nm (OD_600_) of 0.5 was obtained. Aliquots were prepared with a concentration of 5 × 10^6^ cfu/100 µL of *S. aureus*, to which TcPaSK (10, 15, 20, 25 µg/mL, equivalent to 2.82, 4.24, 5.65, 7.06 µM), or 25 µg/mL (7.57 µM) TcDef3, or 25 µg/mL (4.85 µM) human defensin hBD-3 (positive control), or H_2_O (negative control) were added. The treatments were incubated for 8 h at 37 °C and labeled with SYBR Green fluorochromes (Invitrogen, Carlsbad, CA, USA) (25 µL of 25× SYBR^TM^ Green I solution in H_2_O) and propidium iodide (Sigma-Aldrich, Saint Louis, MO, USA) (10 µL of 1 mg/mL propidium iodide). Finally, cell death caused by peptides was analyzed by flow cytometry with the BD Facs Verse (Becton Dickinson, Franklin Lakes, NJ, USA) equipment, at the Cell Culture and Flow Cytometry Service of the Central Research Support Facilities of the University of Valencia. Experiments were run in triplicate.

### 2.7. Scanning Electron Microscopy (SEM)

For SEM analysis, aliquots of the same samples and cell treatments prepared for the antimicrobial activity assays were used. The samples were incubated at 37 °C for 1 h. The bacteria were fixed with Karnovsky’s fixative (2.5% paraformaldehyde and 0.5% glutaraldehyde) for 2 h at 4 °C. After washing (centrifugation and removal of the supernatant), cells were fixed with 2% osmium tetraoxide for a further 2 h, washed again and filtered using a 0.2 µm filter. The cells were dehydrated in graduated series of ethanol (30°, 50°, 70°, 90°, 100°) for 10 min, in each gradation. To perform critical point drying, ethanol was replaced by liquid CO_2_. The samples were shaded with palladium gold for 2 min and the results were observed in the FEG-SEM HITACHI S4800 equipment at 10 Kv, at the Microscopy Service of the Central Research Support Facilities of the University of Valencia.

### 2.8. Transmission Electron Microscopy (TEM)

Samples for TEM analysis were prepared in the same way as for SEM, with the exception of the ethanol graduated dehydration series in which only the 90° gradation was reached. Then, the samples were embedded in resin in 4 steps: 96° ethanol-LR-White 2:1 resin (20 h), 100° ethanol-2:1 resin (20 h), 100° ethanol-1:2 resin (20 h) and 100% resin (24 h at 60 °C). After block preparation, 60 nm-thick ultra-thin sections were cut with a Leica UC6 Ultracut Microtome. The sections were contrasted with lead and the results were observed in the TEM JEOL-JEM1010 equipment at 70 kV, in the Microscopy Service of the Central Research Support Facilities of the University of Valencia.

### 2.9. Cell Viability Assay

The TcPaSK effect on cell viability was determined using the MTS colorimetric assay. Briefly, MDA-MB-231, 4T1 and HC-11 cells were seeded into 96-well flat-bottom cell culture plates and incubated overnight at 37 °C in a humidified atmosphere with 10% CO_2_, at a cell density of 5 × 10^3^ cells/well. Adherent cells were treated with TcPaSK peptide (100, 200, 400, 560, 700 µg/mL) or water and incubated for 24 h at 37 °C. Then, combined MTS/phenazine methosulfate reagent (333 μg/mL MTS and 25 μM phenazine methosulfate) was added and incubated for 2 h at 37 °C in the dark. The absorbance was measured at 490 nm using a microplate reader (BMG-LABTECH, Ortenberg, Germany). Percentage of viability of the TcPaSK treated cells was expressed as percentage compared to control using the formula (1 − E/C) × 100, where E is the absorbance of TcPaSK treated cells and C the absorbance of control cells treated with vehicle (water).

### 2.10. Cell Proliferation Assay

Serum-starved MDA-MB-231 cells were seeded in a 6-well plate and incubated overnight at 37 °C. Adherent cells (1.5 × 10^4^ cells/well) were stained with 1.25 μM Oregon Green 488 dye in warm serum-free DMEM. Following incubation for 45 min at 37 °C, a sample of cells was fixed with 1% [*w/v*] paraformaldehyde to establish baseline fluorescence, and the rest of the cells were treated with TcPaSK (200 μg/mL) or water for 72 h. Then, cells were fixed and analyzed with a FACSCalibur flow cytometer and Cell Quest^TM^ software (version 3.3; BD Biosciences, Mississauga, ON, USA). To calculate the number of cell divisions (n), the following formula was used, MF_baseline_ = MF_treatment_ (2^n^), in which MF denotes the mean fluorescence.

### 2.11. Cell Cycle Analysis

MDA-MB-231 cells were prepared in 6-well plates with a density of 5 × 10^4^ cells per well, to which 200 µL or 400 µL of TcPaSK (final concentrations 100 µg/mL and 200 µg/mL, respectively) were added, or the same volume of medium (solvent with which the dilution of the peptide was performed) in the corresponding controls. The cells were incubated at 37 °C for 72 h. The CycleTEST ™ PLUS DNA Reagent Kit (Sigma-Aldrich, Saint Louis, MO, USA) was used to analyze the cell cycle. Two washings with citrate buffer (sodium citrate, sucrose and dimethyl sulfoxide (DMSO)) were performed, centrifuging the samples in each of them for 5 min at 300× *g*, and a final wash, in which the number of cells was adjusted at 5 × 10^5^ and centrifuged for 5 min at 400× *g*. Next, 250 µL of solution A (trypsin in a spermine tetrahydrochloride detergent buffer), 200 µL of solution B (trypsin and ribonuclease A inhibitor in a citrate stabilizing buffer with spermine tetrahydrochloride) and 200 µL of solution C were added (IP and the citrate stabilizing buffer with spermine tetrahydrochloride), leaving between the addition of each solution an interval of 10 min at room temperature and performing the last one, before filtering, in a cold chamber and in the dark. The analyses of the phase of the cell cycle in which the cells were found were carried out with a BD Facs Verse (Becton Dickinson, Franklin Lakes, NJ, USA) equipment. The cell cycle modeling was performed using Modfit LT software, version 3.3.11. The experiments were performed in triplicate.

### 2.12. Sample Preparation for Proteomic Analyses

Proteins from cell lines were extracted in lysis buffer (7 M Urea, 2 M thiourea, 4% CHAPS). Quantification was done according to RC_DC (BioRad, Hercules, CA, USA) instructions. Fifty μg of a mix from each condition protein extracts (pool C and treated) were mixed with Laemmli sample buffer 1X and denatured for 5 min at 95 °C. The sample was loaded into 1D_SDS_PAGE (Bio-Rad 12%™ Mini-PROTEAN^®^ TGX™ #456-1044) to visualize the protein profile and to clean the protein extract of contaminants. 1D_SDS_PAGE gel was stained with colloidal Coomassie to visualize proteins. An aliquot of mixed samples was introduced into 1D_SDS_PAGE (Any kd BIORAD, Hercules, CA, USA) to generate a library for protein characterization. Twenty µg of each sample were introduced into 1D_SDS_PAGE to perform in gel the digestion procedure for LC_MSMS analysis. For LC_MSMS analysis, the gel fraction was cut and the sample was digested with sequencing grade trypsin (Promega, Madison, WI, USA) as described elsewhere [22]. One μg of trypsin in 150 μL of ABC solution was used. The digestion was stopped with TFA (1% final concentration), a double extraction with ACN was done, and all the peptide solutions were dried in a rotatory evaporator. Samples were re-suspended with 25 μL of 2% ACN; 0.1% TFA for samples and 200 μL for the library.

### 2.13. Library LC-MS/MS Analysis

Five μl of the sample library were loaded onto a trap column (NanoLC Column, 3 μm C18-CL, 75 µm × 15 cm; Eksigen) and desalted with 0.1% TFA at 3 μL/min during 5 min. The peptides were loaded onto an analytical column (LC Column, 3 μm C18-CL, 75 µm × 12 cm, Nikkyo, Tokio, Japan) equilibrated in 5% acetonitrile (ACN) 0.1% FA (formic acid). Peptide elution was carried out with a linear gradient of 5 to 35% B in (A: 0.1% FA; B: ACN, 0.1% FA) for 240 min at a flow rate of 300 nL/min. Peptides were analyzed in a mass spectrometer nanoESI qQTOF (5600 TripleTOF, ABSCIEX, Alcobendas, Spain). Eluted peptides were ionized applying 2.8 kV to the spray emitter. Analyses were carried out in a data-dependent mode (DDA). Survey MS1 scans were acquired from 350–1250 m/z for 250 ms. The quadrupole resolution was set to ‘UNIT’ for MS2 experiments, which were acquired 100–1500 m/z for 150 ms in ‘high sensitivity’ mode. The following switch criteria were used: charge: 2+ to 5+; minimum intensity; 70 counts per second (cps). Up to 25 ions were selected for fragmentation after each survey scan. Dynamic exclusion was set to 15 s. The rolling collision energies equations were set for all ions as for 2+ ions, according to the following equations: |CE| = (slope) × (m/z) + (intercept). ProteinPilot default parameters were used to generate peak list directly from 5600 TripleTof wiff files. The Paragon algorithm of ProteinPilot was used to search Expasy protein database with the following parameters: trypsin specificity, cys-alkylation, *Homo sapiens* taxonomy restrictions, and the search effort set to through and FDR correction. To avoid using the same spectral evidence in more than one protein, the identified proteins were grouped based on MS/MS spectra by the Protein-Pilot Progroup algorithm. Thus, proteins sharing MS/MS spectra were grouped, regardless of the peptide sequence assigned. The protein within each group that can explain more spectral data with confidence was shown as the primary protein of the group. Only the proteins of the group for which there was individual evidence (unique peptides with enough confidence) were also listed, usually toward the end of the protein list. Proteins were identified with an FDR of 1% with taxonomy restriction (unused corrected = 1.010).

### 2.14. SWATH LC-MS/MS Analysis

Samples were acquired in a random way. Treated 3/Control 1/Treated 4/Control 3/Control 4/Treated 2/Control 2/Treated 1. Five μL of each sample were loaded onto a trap column (NanoLC Column, 3μm C18-CL, 75 µm × 15 cm; Eksigent) and desalted with 0.1% TFA at 3 μL/min during 5 min. The peptides were loaded onto an analytical column (LC Column, 3 μm C18-CL, 75 µm × 12 cm, Nikkyo) equilibrated in 5% acetonitrile 0.1% FA (formic acid). Peptide elution was carried out with a linear gradient of 5 to 35% B in (A: 0.1% FA; B: ACN, 0.1% FA) for 180 min at a flow rate of 300 nL/min. Peptides were analyzed in a mass spectrometer nanoESI qQTOF (5600 TripleTOF, ABSCIEX). The tripleTOF was operated in swath mode (independent data analysis), in which a 0.050-s TOF MS scan from 350–1250 m/z was performed, followed by 0.080-s product ion scans from 350–1250 m/z on the defined windows (3.05 s/cycle). Eluted peptides were ionized by applying 2.8 kV to the spray emitter. The tripleTOF was operated in swath mode, in which a 0.050-s TOF MS scan from 350–1250 m/z was performed, followed by 0.080-s product ion scans from 350–1250 m/z on the 32 defined windows (3.05 s/cycle). The rolling collision energies equations were set for all ions as for 2+ ions, according to the following equations: |CE| = (slope) × (m/z) + (intercept).

### 2.15. SWATH Data Analysis and Protein Quantitation

The wiff files obtained from SWATH-MS experiment were analyzed by Peak View 2.1. A total of 8 samples (2 groups, n = 4/group) were analyzed in Expasy (SIB, Switzerland) using SwissProt database and 1571 proteins were quantified. The quantitative data obtained by Peak View were analyzed with Marker View 1.3. PCA aggrupation without protein areas normalization was visualized. Then, areas were normalized by total areas summa, and a PCA analysis was visualized. DA (discriminant analysis) aggrupation was performed. A *t*-test was performed between conditions (control vs. treated) for statistical analysis. 

## 3. Results

### 3.1. TcPaSK Is a Cationic Peptide that Shares the Common Structural Features of AMPs and May Form an Amphipathic α-Helix

In a previous work, antimicrobial activity of a synthetic peptide (Tcdef3) derived from defensin 3 of the *Tribolium castaneum* beetle was reported. This 29-amino acid peptide displayed activity against the human microbial pathogens *Escherichia coli*, *S. aureus* and *Candida albicans*, with *S. aureus* being the most susceptible one [19]. 

The amino acid residues that flank Tcdef3 fragment in the sequence of *T. castaneum* defensin 3 are lysine and asparagine at the N- and C-terminus, respectively. On the basis of the theoretical predictions of the physicochemical parameters that mainly determine the antimicrobial activity (charge, amphipathicity and hydrophobicity), we compared the properties of Tcdef3 peptide with those of an extended peptide (TcPaSK) that contained the two amino acid residues that flank Tcdef3 fragment in the defensin 3 sequence. The primary peptide sequence and prediction scores of Tcdef3 and TcPaSK peptides are summarized in Table 1.

The general strategy of increasing the cationicity and keeping moderate hydrophobicity of the AMP has been found to be very effective in improving antimicrobial activity [23,24]. According to this approach, the charge and isoelectric point of the parental peptide increased in TcPaSK (due to the addition of the lysine residue at the N-terminus), whereas the hydrophobic ratio decreased (due to the uncharged polar asparagine residue at the TcPaSK C-terminus), theoretically enhancing its ability to target and interact with negatively charged components of microbial membranes. Based on the bacterial origin of mitochondria in eukaryotic cells, it would be expected that TcPaSK might also interact with mitochondrial membranes.

**Figure 1:**
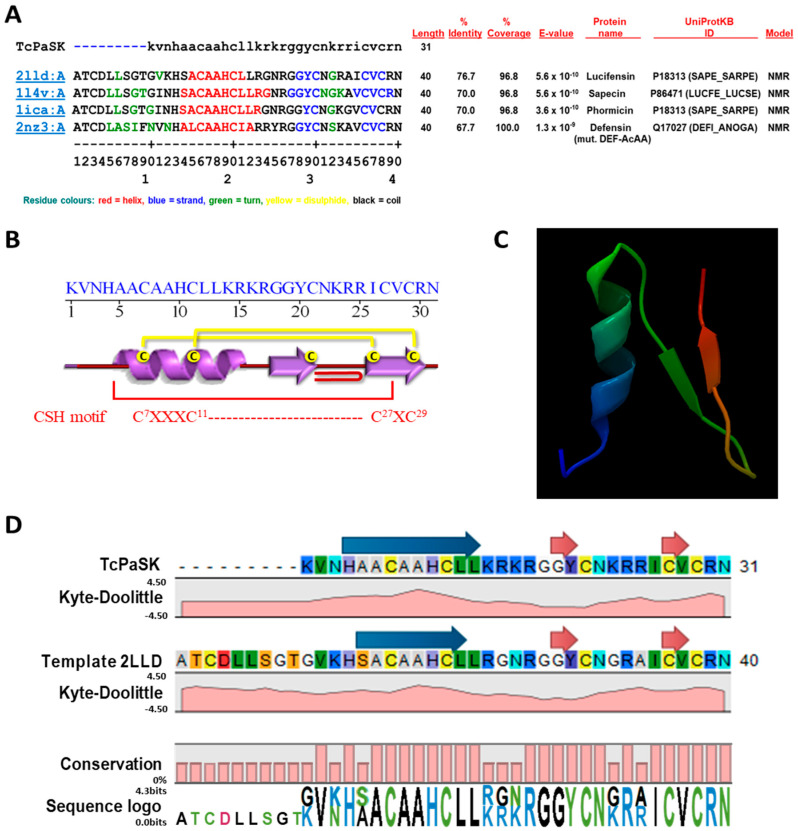
Structural organization, properties and 3D modelling of TcPaSK peptide. (**A**) Multiple sequence alignment of the TcPaSK peptide sequence using Sequence Annotated by Structure (SAS) tool (https://www.ebi.ac.uk/thornton-srv/databases/sas/ [25]) that performs a FASTA search against the PDB database (http://www.rcsb.org/). Alignment was used for template identification. On the basis of the template properties obtained from CLC genomics workbench v 20 analysis, 2LLD sequence was selected as reference for the 3D-modelling of TcPaSK structure. (**B**) Schematic representation of the secondary structure of the TcPaSK peptide. The α-helix is represented by a purple spiral ribbon and the β-strands by purple arrows. The different structural motifs, including a β-hairpin (represented by ⊃) and a CHS motif, are indicated. Disulphide bridges are represented as yellow connecting lines. (**C**) Modelling of the 3D-structure of TcPaSK peptide using SWISS-MODEL software (http://swissmodel.expasy.org) using 2LLD as selected template. (**D**) Comparison of Kyte–Doolittle regional hydropathy prediction of TcPaSK and 2LLD template, and generation of the sequence logo based on the conservation of the target-template alignment, using the CLC genomics workbench v 20. Blue arrows indicate a predicted α-helix region and red arrows correspond to predicted β-strands.

Secondary structure analysis of TcPaSK peptide was performed using the sequence annotated by structure (SAS) tool that searches a given protein sequence against all Protein Data Bank (PDB) sequences and structurally annotates alignment (https://www.ebi.ac.uk/thornton-srv/databases/sas/) [25]. As shown in the FASTA alignment of the amino acid sequence of TcPaSK peptide and the top protein hits within the PDB database (Figure 1A), a similar topological structure for TcPaSK is predicted, including an N-terminal sequence, followed by an α-helix and two antiparallel β-strands forming a so-called ‘cysteine-stabilized α-helix (CSH) motif’ [26,27] that consists of one pair of cysteine residues spaced by a tripeptide sequence (Cys-X-X-X-Cys) in an α-helix (C^7^-A-A-H-C^11^ in TcPaSK) and crosslinked by two disulphide bridges with a fragment Cys-X-Cys in a β-strand (C^27^-V-C^29^ in TcPaSK) (Figure 1B). The CSH motif is found in insect and plant defensins, serine proteinase inhibitors, and some scorpion neurotoxins, to name a few [28]. An atypical β-bulge formed by a G-X-C motif between the third and fourth Cys is also present in TcPaSK peptide (G^19^-Y-C^21^). This motif is a common feature conserved in many mammalian β-defensins, as well as in insect defensins [29,30]. Moreover, TcPaSK peptide contains a conserved tyrosine (Y^29^) placed adjacent to a disulphide bond and forming part of a β-sheet shared between αβ and β-hairpin AMPs [31].

Molecular modelling of the three-dimensional structure of TcPaSK peptide was performed by CLC Genomics Workbench 20 (Qiagen, Hilden, Germany) software using the above-mentioned top four FASTA alignment hits as templates. Quality assessment of TcPaSK peptide structure prediction was based on the composite scoring function QMEAN (Swiss Model platform https://swissmodel.expasy.org/qmean/), which evaluates several structural features of peptides and estimates the likelihood that a given model is of comparable quality to experimental structures [32] (Supplementary Figure S1). Lucifensin chain A (PDB code 2LLD) was used as reference molecule to design, on the basis of its structure, the 3D molecular model of TcPaSK peptide (Figure 1C). Structural properties of the template-based TcPaSK peptide structure modelling are shown in Figure 1D. 

The global α-β fold of the TcPaSK peptide displays the same general features as those predicted for the Tcdef3 peptide [19]. Helical wheel projection of the α-helix region of these peptides showed an amphipathic configuration in which amino acids with non-polar side chains (A^5^, A^6^, A^8^, A^9^, L^12^ and L^13^) are spatially concentrated on the hydrophobic face of the predicted α-helix and hydrophilic residues are located on the opposite side (Supplementary Figure S2).

In summary, TcPaSK is a short cationic peptide that shares the common structural features of AMPs and may form amphipathic α-helices that enable the peptide to interact with membranes and, as with Tcdef3 peptide, may act as an antimicrobial peptide. In addition to the higher cationicity of TcPaSK peptide, according to the theoretical predictions of the physicochemical and structural properties, the main difference between TcPaSK and Tcdef3 peptides is the N residue at the C-terminus that reduces the hydrobophicity of TcPaSK and may improve its antimicrobial activity, as proposed for other AMPs [33,34].

### 3.2. TcPaSK Peptide Exhibits Potent Antibacterial Activity against S. aureus

*S. aureus* was the most susceptible microorganism of those tested with Tcdef3 [19]. TcPaSK peptide antimicrobial activity was estimated by measuring the MIC value. Results showed that TcPaSK was effective against *S. aureus* cells at a MIC ranging from 16 to 32 µg/mL (Supplementary Table S1). 

The hemolytic activity of TcPaSK peptide against human RBC was determined at a concentration range of 2.35–75 μM (Supplementary Table S2). Melittin, used as control hemolytic peptide, induced 100% hemolysis at 1.25 μM, whereas TcPaSK caused 7.28 ± 0.1% hemolysis at concentration of 2.35 μM (corresponding to 10 μg/mL).

**Figure 2:**
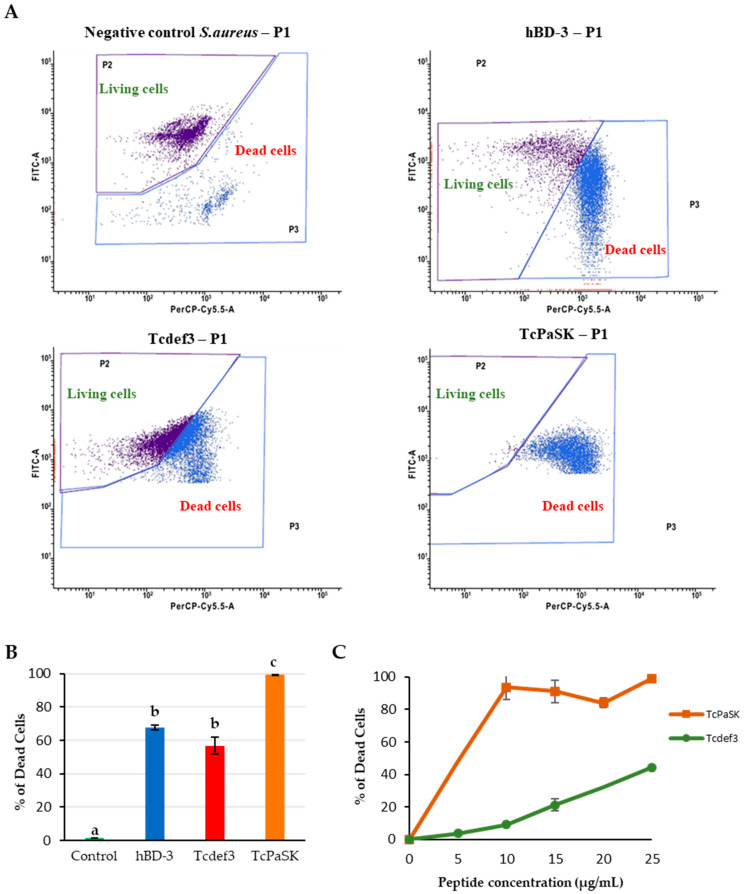
Antibacterial activity of hBD-3, Tcdef3 and TcPaSK peptides against *S. aureus*. (**A**) Flow cytometry dot plots of *S. aureus* cells non-treated (negative control) and treated with hBD-3 (positive control), Tcdef3 and TcPaSK peptides, stained with SYBR Green (membrane permeable) and propidium iodide (membrane impermeable) dyes. On the ordinate, FICT refers to fluoroscein and indicates the cells that have incorporated SYBR Green. On the abscissa axis, PerCP-Cy5.5-A refers to Peridinin-chlorophyll proteins and corresponds to cells that have taken up propidium iodide. (**B**) Cytotoxicity against *S. aureus* of 25 µg/mL hBD-3, Tcdef3 and TcPaSK peptides. Data represent the mean ± SE of three replicates. Bars with different letters indicate statistically significant differences between AMPs (Student’s *t*-test, *p* ≤ 0.05). (**C**) Effect of Tcdef3 and TcPaSK peptide concentration on *S. aureus* cells viability. The results are expressed as the percentages of the total death cells after 1 h incubation time with the peptides. Points are the means ± SE of two replicates.

We evaluated the antibacterial activity of TcPaSK peptide against *S. aureus* (Figure 2) by flow cytometry using a SYBR Green/propidium iodide (PI) double staining assay (SYBR Green I stains all cells, whereas PI stains only the nucleic acids in cells when their membranes are disrupted). As shown in Figure 2A, 1.58% of *S. aureus* cells were stained with PI in the absence of peptide, and after treatment with 25 μg/mL TcPaSK (within MIC range), Tcdef3 or human defensin-3 (hBD-3, used as positive control at a concentration giving the same mortality level of Tcdef3) 99.11%, 56.88% and 67.70% of cells presented an increase in PI fluorescence signal, respectively. TcPaSK peptide displayed the highest activity, being 43% and 33% more active than Tcdef3 and hBD-3 peptides, respectively (Figure 2B).

We also analyzed the TcPaSK antimicrobial activity against *S. aureus* when peptide concentrations lower than 25 μg/mL were used in the assay. Figure 2C shows that, at a concentration as low as 10 μg/mL, where TcDef3 peptide-treated cell mortality was around 9% [19], TcPaSK cytotoxicity nearly reached 100%. 

### 3.3. TcPaSK Peptide Induces Morphological Alteration of S. aureus Cells

**Figure 3:**
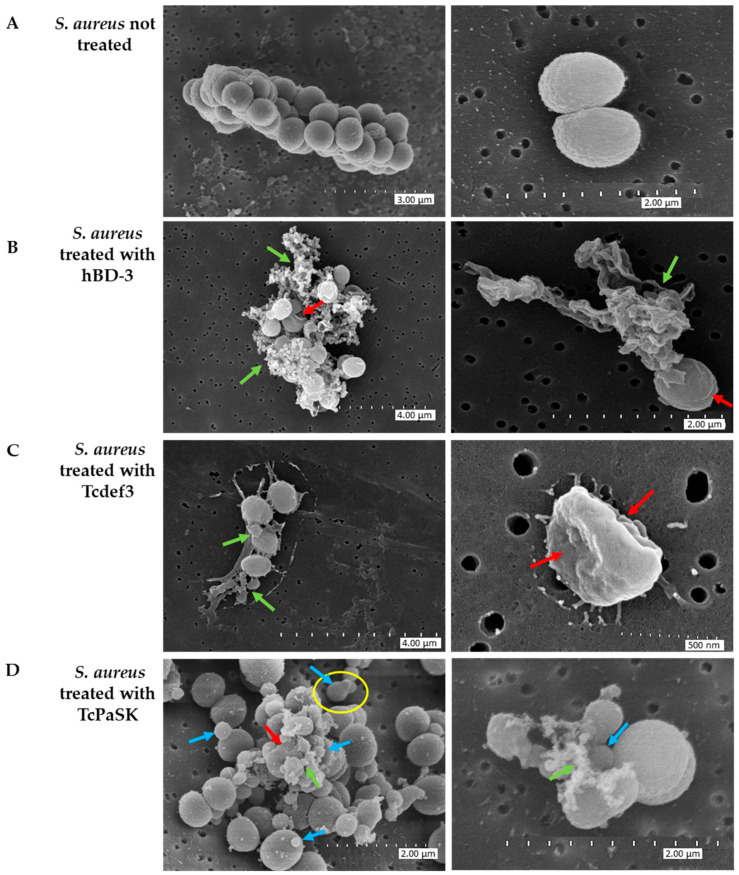
SEM micrographs of *S. aureus* cells treated with 25 µg/mL hBD-3, Tcdef3, or TcPaSK peptides during 1 h. (**A**) non-treated control, (**B**) hBD-3 peptide, (**C**) Tcdef3 peptide, and (**D**) TcPaSK peptide. Red arrows indicate ruptures and bulges in the membrane, green arrows cytoplasmic debris, and blue arrows blebs and ghost cells. The yellow circle highlights a dividing cell.

*S. aureus* cellular damage induced by TcPaSK peptide was visualized by SEM (scanning electron microscopy), which provides high resolution images of the cell morphology. Figure 3 shows the SEM images of *S. aureus* CECT 4013 cells after 1 h exposure to 25 μg/mL TcPaSK peptide. For comparison, Tcdef3 and hBD-3 peptides were also used. Untreated control cells exhibited bright and smooth surfaces, and intact cellular membrane, whereas peptide treatment induced significant cell morphological changes. Upon hDB-3 treatment, cells became roughened and deformed, and in many cases, cytoplasmic contents were leaking out of the cells (green arrows) from significant disruptions of the cellular membrane (red arrows). Similar to, but less extensive than that with hDB-3 peptide, was the effect on the cell membrane of Tcdef3 peptide treatment (green arrows), in which corrugated and shrunken cells (red arrows) were also detected. Cells treated with TcPaSK peptide displayed a distorted appearance, membrane irregular morphologies (red arrows), complete cell rupture with concomitant liberation of cytoplasmic contents (green arrows), and blebs and ghost cells (blue arrows). 

### 3.4. TcPaSK Peptide Alters Cell Membrane Integrity and Impairs Cell Division in S. aureus

**Figure 4:**
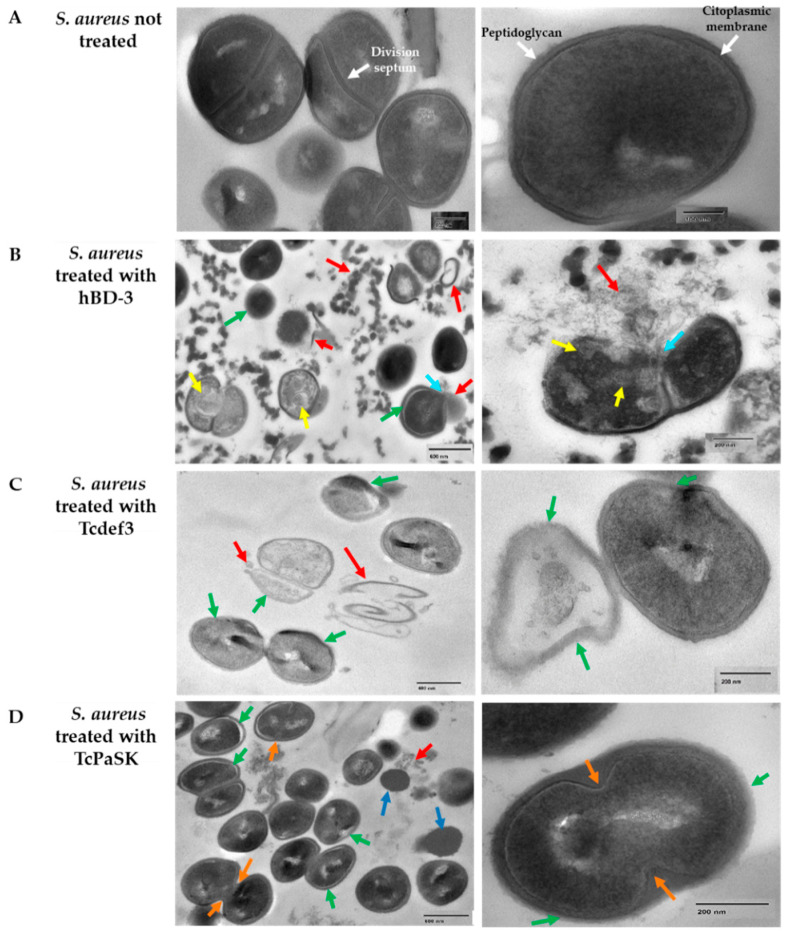
TEM micrographs of *S. aureus* cells were treated with 25 µg/mL hBD-3, Tcdef3, or TcPaSK during 1 h. (**A**) non-treated control, (**B**) hBD-3 peptide, (**C**) Tcdef3 peptide, and (**D**) TcPaSK peptide. Red arrows indicate cytoplasmic and cell wall debris; green arrows cell distortions, membrane shedding, and thinning of peptidoglycan; light blue arrows indicate cell lysis, yellow arrows indicate cytoplasmic vacuolization; orange arrows indicate inhibition of the division septum; and the dark blue arrows indicate highly electrodense structures that only appear in cells treated with the TcPaSK peptide.

TEM (transmission electron microscopy) was employed to investigate the ultrastructural alterations of *S. aureus* cells treated with TcPaSK peptide and to compare with the effect of hDB-3 and Tcdef3 peptides. Figure 4 shows the TEM images of *S. aureus* CECT 4013 cells after treatment with peptides (25 μg/mL concentration of either TcPaSK, Tcdef3 or hBD-3) during 1 h. In untreated control cells, an intact cytoplasmic membrane and the typical petptidoglycan cell wall (white arrows) that characterizes Gram+ bacteria were observed, as well as unaltered division septum (purple arrows). In contrast, treatment with hBD-3 induced cell distortion and membrane rupture (green and blue arrows, respectively), release of cellular contents (red arrows), and cytoplasm vacuolization (yellow arrows). In *S. aureus* cells treated with Tcdef3 peptide, the cytoplasmic membranes became blurred (green arrows) and began to collapse (red arrows), and dispersion of the intracellular contents was observed. Following treatment with TcPaSK peptide, *S. aureus* cells exhibited malformations, irregular plasma membrane occasionally separated from the outer cell membrane (green arrows), leakage of cytoplasmic and cell wall debris (red arrow), and division septum inhibition (orange arrows). The high proportion of cells containing septa in *S. aureus* cells treated with TcPaSK peptide when compared with control samples may be due to TcPaSK peptide interfering with cell division at this stage. In these samples, TEM technique allows observation of the ultrastructure of ghost cells and cells without visible intracellular organelles (dark blue arrows), that exhibited a differential electrodensity and are characterized by a lack of cytoplasmic membrane and peptidoglycan cell wall. 

### 3.5. TcPaSK Peptide Interacts with Mammalian Cells

**Figure 5:**
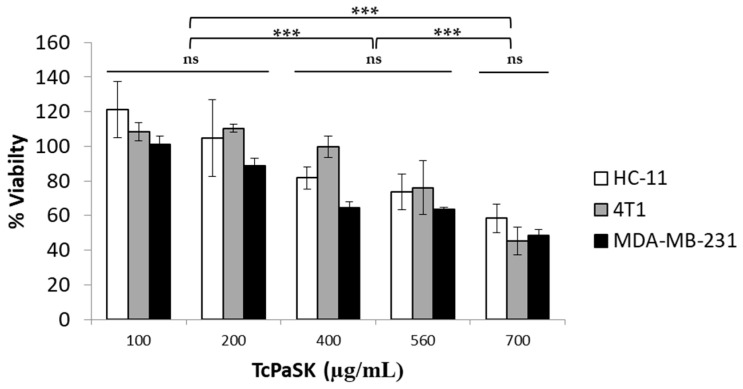
Effect of TcPaSK on mammalian cell viability. Human MDA-MB-231 TNBC cells, 4T1 mouse mammary carcinoma cells and HC11 mouse mammary epithelial cells were cultured in the presence of medium, vehicle (water) or the indicated concentrations of TcPaSK peptide for 24 h. Cell viability was assessed by MTS and results were normalized with respect to cells cultured in medium (with vehicle). Statistical analysis of 4 independent experiments was performed using analysis of variance (ANOVA) and Tukey’s multiple comparisons test; ns: not significant, *** *p* < 0.001.

Some antimicrobial peptides that kill microbial pathogens also exhibit selective anticancer activity and have been considered promising for cancer therapy, either alone or in combination with conventional drugs [13]. To investigate the potential antiproliferative activity of TcPaSK peptide, its effect on the viability of MDA-MB-231 human TNBC cells and 4T1 mouse mammary carcinoma cells, as well as the non-malignant HC11 mouse mammary epithelial cells, was assessed by MTS assays. Results showed that TcPaSK peptide reduced the number of viable cancer cells, as well as viable normal mammary epithelial cells, in a concentration-dependent manner (Figure 5). However, the range of peptide concentrations that exerted a cytotoxic effect on mouse and human cell lines was significantly higher than the range of concentrations in which TcPaSK kills *S. aureus* cells. Whereas, at a peptide concentration of 10 µg/mL nearly 100% *S. aureus* cell death was observed (Figure 2), no effect in mammalian cells’ viability was detected at 100 µg/mL peptide concentration (Figure 5), which gives a broad safety margin for the use of TcPaSK in antimicrobial therapies.

### 3.6. TcPaSK Peptide Inhibits MDA-MB-231 TNBC Cell Proliferation at a Subcytotoxic Concentration by Blocking G1-S Cell Cycle Progression

**Figure 6:**
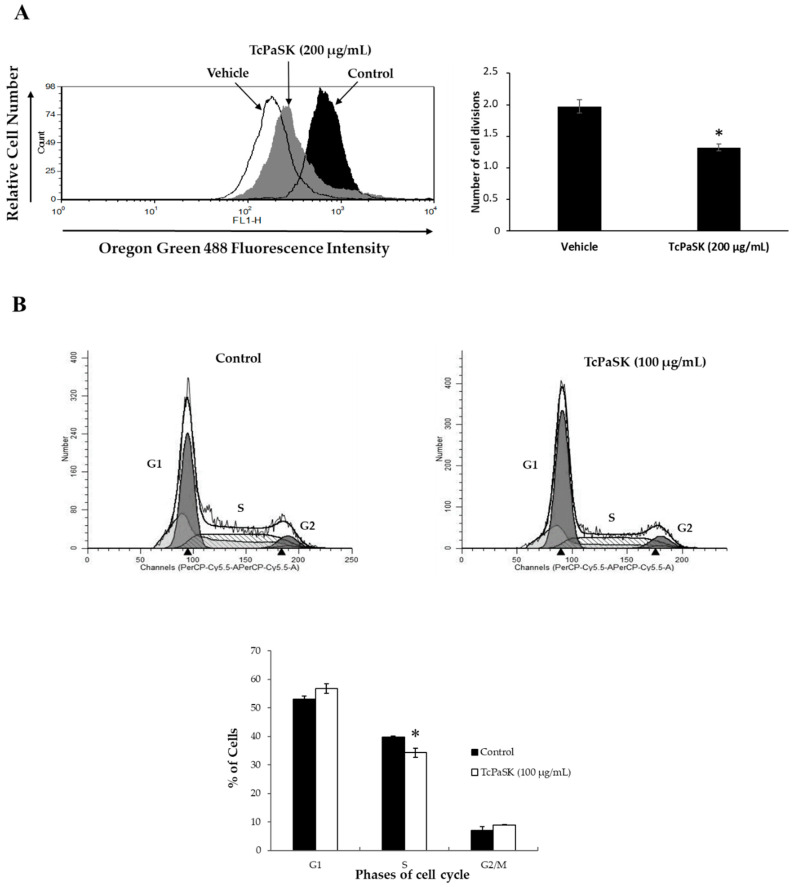
Antiproliferative effect of TcPaSK on MDA-MB-231 TNBC cells. (**A**) MDA-MB-231 TNBC cells were stained with Oregon Green 488 dye and cultured in the presence of medium, vehicle (water) or 200 µg/mL TcPaSK for 72 h. Oregon Green-488 fluorescence was quantified by flow cytometry as a measure of cell proliferation and representative data are shown. The number of cell divisions was calculated, and data shown are the mean of at least 3 independent experiments ± SE. Asterisk indicates that differences between means of non-treated and TcPaSK-treated cells were statistically significant (Student’s *t*-test, *p* < 0.05). (**B**) MDA-MB-231 cells were cultured for 72 h in the absence or presence of 100 µg/mL TcPaSK peptide, then stained with PI and analyzed by flow cytometry to determine cell distribution among three major phases of the cell cycle (G1, S and G2/M). Representative histograms are shown. Data represent the mean ± SE of at least 3 independent experiments. Asterisks indicate that differences between means of non-treated and TcPaSK-treated cells were statistically significant (Student’s *t*-test, *p* < 0.05).

Although potential toxicity to mammalian healthy cells may pose a limitation for the use of AMPs in cancer therapy, at subcytotoxic doses, AMPs have proved useful as chemosensitizing agents in combined therapies with other conventional anticancer drugs [35,36]. To explore whether TcPaSK peptide could be used as anticancer agent to treat TNBC at concentrations that are harmless for normal cells, we assessed the antiproliferative activity of TcPaSK peptide at a concentration of 200 µg/mL, that only caused a very slight effect on MDA-MB-231 TNBC cell viability and had no effect on the mouse cell lines analyzed (Figure 5). We used the collagen-binding fluorescent dye, Oregon Green 488, the fluorescence of which is halved with each cycle of cell division, to measure cell division. Mean fluorescence (MF) of vehicle (water)- and TcPaSK-treated MDA-MB-TNBC cells of two independent experiments were recorded and normalized using fluorescence data of the non-proliferative fixed cells to determine the number of cycles of cell divisions that occurred. As shown in Figure 6A, the number of cell divisions was significantly decreased by TcPaSK when compared to the water-treated control MDA-MB-231 TNBC cells (33% reduction in rounds of cell division in comparison to vehicle control, *p* < 0.05). To further study the antiproliferative properties of TcPaSK peptide at sublethal concentrations, cell cycle analysis of MDA-MB-231 cells following 72 h exposure to 100 µg/mL TcPaSK was carried out, which did not affect cell viability as assessed by MTS assays (Figure 5). Treatment with TcPaSK caused a significant decrease in the % number of cells in S phase compared to water-treated cells (33.16 ± 1.59%, *p* < 0.05) (Figure 6B), suggesting that TcPaSK peptide may arrest replication of MDA-MB-231 cells at G1/S at a sublethal dose.

### 3.7. TcPaSK Affects MDA-MB-231 TNBC Cell Expression of Proteins Involved in Cell Growth and Tumor Progression

**Figure 7:**
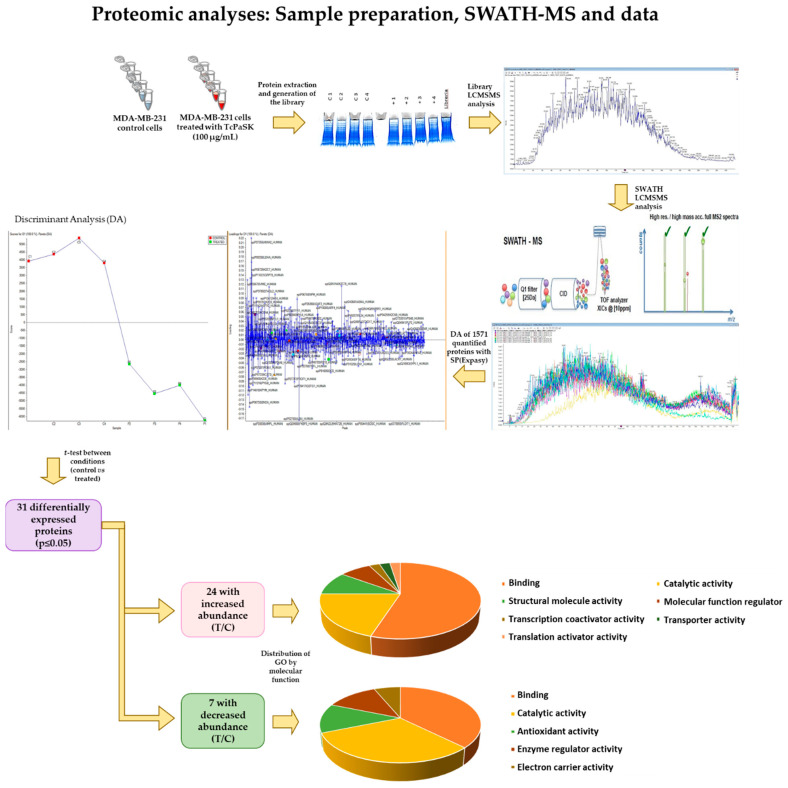
Quantitative SWATH proteomic analysis. The workflow indicates the steps followed in the SWATH analysis performed on four biological replicates of non-treated MDA-MB-231 cells treated and four biological replicates of MDA-MB-231 cells treated with 100 µg/mL TcPaSK peptide. Out of the 1571 proteins identified by means of bioinformatics analysis, 31 were differentially expressed in treated samples as compared to non-treated samples (*t*-test, *p* ≤ 0.5). GO molecular function categories of increased and decreased abundance proteins (Treatment vs. Control) are shown.

To further investigate the impact of a sublethal concentration of TcPaSK on MDA-MB-231 cells, SWATH-MS based quantitative proteomics analysis was performed on eight samples, 4 replicates of MDA-MB-231 TNBC control cells and 4 replicates of MDA-MB-231 cells treated with 100 µg/mL TcPaSK for 72 h (Figure 7). To build the spectral library needed for the SWATH-MS analysis, a peptide mixture of control and TcPaSK treated cells was run using a data-dependent acquisition (DDA) method. The integrated DDA data sets were searched against the *Homo sapiens* UniProt fasta database by using Protein Pilot^TM^ at a 1% critical false discovery rate (FDR) and a total of 1571 proteins were successfully identified. Marker View^®^v1.3 (AB Sciex, Alcobendas, Spain) was used to analyze the quantitative data obtained and the discriminant analysis (DA) performed evidenced distinct clustering of the four control and TcPaSK treatment samples (Figure 7). Table 2 shows the identified proteins exhibiting a relative change in protein ratio in treatment samples vs. control samples with a corrected *p*-value inferior to 0.05. Twenty-four proteins were found downregulated, and seven proteins were upregulated in MDA-MB-231 cells treated with TcPaSK peptide compared to water-treated control cells (Table 2). Both within the downregulated proteins, as well as within the upregulated proteins in TcPaSK treated cells, the largest GO molecular function categories were binding proteins (91.7% and 85.7%, respectively) followed by catalytic activity (33.3% and 71.4%, respectively) (Figure 7). Highlighted in bold in Table 2 are proteins displaying ≥ 1.5-fold change, of which fourteen proteins were found downregulated (45.1% of the total number of differentially expressed proteins, *p* ≤ 0.05), and three proteins were upregulated (9.6% of the total number of differentially expressed proteins, *p* ≤ 0.05) in MDA-MB-231 cells treated with TcPaSK peptide, compared to non-treated control cells. All of them were found to be present in MDA-MB-231 cells when searched in the proteomic landscape of TNBC database (https://zucchini.gs.washington.edu/BreastCancerProteome/; [37]), as well as in other different types of tumor tissues when searched in the human protein atlas database (https://proteinatlas.org; [38]). Moreover, the overexpression of several of these proteins has been specifically correlated with disease progression and adverse prognosis as with La-related protein 1 (LARP1) in cervical and non-small cell lung cancers [39], density-regulated protein (DENR) in hepatocellular carcinoma, gastric cancer, kidney cancer, laryngeal cancer and lung cancer [40], U2AF2 splicing factor in prostate cancer [41], adenylate kinase 2 (AK2) protein in pulmonary adenocarcinoma [42], DNA polymerase delta catalytic subunit in breast cancer (POLD1) in breast cancer [43], endophilin A in basal-like breast cancers [44], thioredoxin reductase (TRXR1) in hepatocellular carcinoma [45], matrix metalloproteinase 14 (MMP14) in colorectal cancer [46] and gluthatione peroxidase 1 (GPX1) in oral squamous cell carcinoma [47].

## 4. Discussion

Invertebrate defensins are host defense peptides involved in antibacterial, antifungal, antiviral, innate immune and inflammation responses [14,48]. Among them, insect defensins containing the cysteine-stabilized αβ (CS-αβ) motif belong to the superfamily of proteins known as scorpion toxin-like superfamily in the SCOP2 database [49]. CS-αβ fold insect defensins are cysteine-rich small peptides (34 to 54 amino acid residues) that adopt an α-helix and two or three β-strands conformation which is stabilized by three or four disulphide bonds [50]. Two of the bonds correspond to a conserved structural motif, the cysteine-stabilized α-helix (CSH) motif (Cys-X-Cys and Cys-X-X-X-Cys) [51].

In an earlier study, a 29-amino acid peptide (Tcdef3) derived from defensin 3 of the *T. castaneum* beetle was synthesized and we reported its antimicrobial activity against the human microbial pathogens *Escherichia coli, S. aureus* and *Candida albicans* [19]. Tcdef3 exhibited the highest level of antimicrobial activity against *S. aureus,* bacteria that are a major cause of several difficult-to-treat infections that seriously threaten human health. In the current work, on the basis of the physicochemical and structural antimicrobial properties of AMPs, we designed a Tcdef3 extended peptide (TcPaSK) (Table 1), derived from the parental sequence of *T. castaneum* defensin 3, having higher antibacterial activity against *S. aureus* (Figure 2). The 31-amino-acid TcPaSK peptide contained the two amino acid residues that flank Tcdef3 fragment in defensin 3 sequence, Lysine at the N-terminus and Asparagine at the C-terminus. Lysine enhanced cationicity and *pI*, and asparagine resulted in decreased hydrobophicity of TcPaSK (Table 1), essential physicochemical determinants that may improve its antimicrobial activity, as proposed for other AMPs [14]. The presence of K at the N-terminus of TcPaSK peptide not only increases the total positive charge that often results in greater affinity for the microbial membrane and enhanced antimicrobial activity, but also, residues at the N-terminal loop are both proposed to be involved in the modulation of the activity and the toxicity of AMPs [23]. The higher *pI* of TcPaSK favors aqueous solubility and prevents the self-aggregation of the molecule in a solution that otherwise would cause it to lose its ability to interact with the cell membrane.

In addition to the peptide charge and hydrophobicity, amphipathicity is the most important sequence-dependent peptide feature, since it allows antimicrobial peptides to pass across cell membranes without requiring a transport system, by interacting with the amphiphilic structure of the lipids [31]. In addition to a typical CS-αβ fold, the 3D modelling of the TcPaSK peptide (Figure 2C) predicted the presence of one amphipathic α-helix, in which hydrophobic residues are placed on one face of the helix and hydrophilic residues on the other (Figure S1). The amphipathic character of AMPs plays a crucial role for their incorporation into the hydrophobic core of the membrane lipid bilayer. This structure enables TcPaSK peptide to interact with the cell membrane, which can then lead to membrane permeation. 

TcPaSK peptide’s antimicrobial activity was significantly higher than that of Tcdef3 and hBD3 peptides (Figure 2). The ability of the TcPaSK peptide to disrupt the cell membrane was further confirmed by SEM and TEM analyses (Figure 3 and Figure 4). Distorted appearance, depressions, blebs and membrane ruptures leading to leakage of cellular contents were observed in *S. aureus* cells challenged with TcPaSK peptide, suggesting that it may exert its toxic action primarily via membrane disruption. A high proportion of *S. aureus* cells treated with TcPaSK peptide were found to contain septa, compared with the control samples. This observation may be also indicative of a TcPaSK peptide mechanism-of-action involving the inhibition of cell division [52]. 

In addition to displaying membrane-lytic activities, AMPs that can also act as anticancer peptides have been described to affect multiple intracellular targets, and thus might confer an advantage in comparison to conventional monotherapies regarding the slower development of chemoresistance [13]. In the case of TNBC, characterized by poor prognosis and limited treatment options due to the lack of molecular targets, development of drug resistance has become a major challenge. Therefore, finding novel combination therapies that increase response rates and circumvent drug-induced resistance is an evolving field in the therapeutic landscape of TNBC. In this context, combination therapies that include AMPs with anticancer activity might be of interest, and for this reason, in the present paper, we have also analyzed the antiproliferative activity of the TcPaSK peptide on MDA-MB-231 TNBC cells.

Our results demonstrated that TcPaSK peptide interaction with mammalian cells decreased cell viability at high concentrations (Figure 5), but at sublethal doses, exhibited antiproliferative activity against MDA-MB-231 cells by blocking G1-S cell cycle progression (Figure 6). The range of TcPaSK peptide concentrations needed for effective antimicrobial activity against *S. aureus* or inhibition of cell proliferation in mammalian cells are significantly different, supporting a safe therapeutic use of this peptide in different pathological contexts. Toxicity is a major hurdle for the development of AMPs as drugs, as it may severely compromise their clinical utility, so understanding the mechanisms by which different AMPs exert their action and their effects on the human body is a requirement for the design and construction of novel AMPs with increased efficacy and reduced undesirable properties [53]. Moreover, exploring all the potential applications of AMPs in different therapeutic fields may turn them into versatile therapeutic agents, worthy of investment from the point of view of the pharmaceutical industry.

The SWATH comparative proteomic analysis of MDA-MB-231 cells treated with TcPaSK peptide against non-treated cells carried out in the present work provided interesting insights into the potential cellular mechanisms underlying the antiproliferative effect of sublethal doses of TcPaSK peptide on MDA-MB-231 cells. Out of thirty-one proteins found significantly differentially expressed (*p* ≤ 0.5) in TcPaSK treated cells compared to non-treated cells, seventeen proteins exhibited ≥1.5-fold down or upregulation (fourteen and three proteins, respectively, Table 2). All of these proteins were previously reported to be present in TNBC cells and other cancer types, and in many cases, correlating with adverse prognosis. 

Regarding downregulated proteins, remarkably, a number of reports described the inhibition of cell growth and/or tumor cell invasiveness, cell cycle arrest, or increased apoptosis upon reduced expression by downregulation or silencing of eleven out of the fourteen protein targets identified in the present work exhibiting ≥1.5-fold decrease expression in TcPaSK treated cells compared to non-treated control cells. Additionally, five of the fourteen protein targets displaying significantly lower abundance upon TcPaSK peptide treatment were previously reported to sensitize cancer cells to radiation and/or conventional anticancer drugs following knockdown or downregulation. Reduced ATPase ASNA1 expression was associated with the significant inhibition of cell growth, increased apoptosis and increased sensitivity to cisplatin and arsenite in human melanoma cells [54] and increased sensitivity to cisplatin, carboplatin, oxaliplatin and arsenite in human ovarian cancer cells [55]. Uroporphyrinogen decarboxylase (UROD) downregulation induced caspase-mediated apoptosis and cell cycle arrest in head and neck cancer cells and radiosensitized several different models of human cancer, increasing tumor cell sensitivity to chemotherapeutic agents, including 5-fluorouracil, cisplatin, and paclitaxel [56]. RNA helicase associated with AU-rich element (DHX36) knockdown resulted in significant increases in PTX1 protein levels, a transcription factor, with roles in development and as a tumor-suppressor [57]. LARP1 silencing inhibited cell migration in prostate cancer [58] and cell proliferation in colorectal cancer [59], and sensitized ovarian cancer cells to cisplatin, paclitaxel and gemcitabine [60]. U2AF2 knockdown significantly suppressed melanoma cell motility [61]. The silencing of the tyrosine protein kinase BAZB1 reduced melanoma tumor growth [62]. The downregulation of transmembrane 9 superfamily member 4 (TM9SF4), a positive regulator of V-ATPase activity, significantly inhibited tumor cell invasiveness and increased the cytotoxic effect of 5-FU in colon cancer cells [63]. POLD1 downregulation suppressed cell proliferation and cell cycle progression in breast cancer cells [43]. Knockdown of AK2 suppressed proliferation, migration, and invasion as well as induction of apoptosis and autophagy in human lung adenocarcinoma cells, and even greater tumor suppression was observed when AK2 silencing was combined with hydroxychloroquine, an effective autophagy inhibitor [42]. Depletion of 60 S ribosomal protein L5 (RPL15) caused ribosomal stress, resulting in apoptosis in colon cancer cells [64]. Endophilin A2 silencing caused a significant reduction in TNBC tumor growth and lung metastasis [44]. 

Our results also showed that upon peptide treatment, three proteins reported to sustain high proliferation rates in cancer cells (TRXR1, MMP14 and GPX1) were upregulated in MDA-MB-231 cells. GPX1 and TRXR1 are intracellular antioxidant enzymes that play a role supporting cancer progression, by counteracting substantial amounts of reactive oxygen species (ROS) generated in tumor cells [65]. However, it has been proposed that the overall redox state of the cell influences whether excess of antioxidant enzymes is pro- or anti-survival, since in excess, antioxidant enzymes may also have deleterious effects, due to the lack of essential cellular oxidants that can cause reductive stress and diminish cell growth responses [66]. MMP14 is a matrix metalloproteinase that regulates the activity of multiple extracellular and plasma membrane proteins, influencing cell–cell and cell–extracellular matrix (ECM) communication, thereby mediating processes such as extracellular matrix degradation and remodeling, cell invasion, and cancer metastasis [67]. Nevertheless, MMP14 activity is also modulated by other proteins, and interestingly, it has been reported that endophilin A2 knockdown in TNBC cells provoked defects in three-dimensional cell invasion, and this correlated with impaired extracellular matrix degradation and the internalization of MMP14 [44]. Endophilin A2 is one of the proteins identified in the present study that was downregulated by TcPaSK peptide in MDA-MB-231 cells, and this effect could counteract MMP14 upregulation. Other interactions and compensations might also be taking place among the cellular processes in which the identified proteins with impaired expression are involved in rendering the observed reduced cell proliferation as the final outcome.

In recent years, increasing evidence has demonstrated the ability of AMPs to target intracellular processes in bacteria, resulting in a myriad of inhibitory activities that range from nucleic acids or protein biosynthesis, and protein-folding inhibition to protease, cell division, cell wall biosynthesis, or lipopolysaccharide inhibition. The study of these multifunctional AMPs might be relevant for the development of novel antibacterial agents with improved therapeutic profiles that are less prone to promote the development of resistance [68]. Natural human AMPs also have a role in different human conditions, including infectious and autoimmune diseases, and cancer [69], and have been taken as a reference to analyze AMPs’ structure-function relationships and their impact in health and disease. Our proteomic analysis highlights the potential antitumor activity of sublethal concentrations of TcPaSK peptide, based on the downregulation of multiple cellular targets. Given that several of the downregulated proteins can sensitize cancer cells to conventional chemotherapy and/or radiotherapy when present in lower amounts, the suitability of including this novel therapeutic peptide in combination with chemotherapeutic agents, such as doxorubicin or cisplatin, with different modes of action and that may show synergistic anti-tumor effects remains to be explored. Further characterization of the mechanisms by which TcPaSK cell membrane interaction alters the expression of the proteins identified in the present study and exerts its antiproliferative activity in MDA-MB-231 cells might shed light into novel pathways and targets to attack TNBC.

## 5. Conclusions

In the present work, we have analyzed the antimicrobial activity against *S. aureus* of the TcPaSK peptide derived from the *T. casteneum* insect defensin 3. Our results showed a significantly high antimicrobial activity due to its ability to disrupt the bacterial cell membrane confirmed by SEM and TEM analyses. In addition, sublethal concentrations of TcPaSK peptide had an antiproliferative effect on MDA-MB-231 TNBC cells by blocking G1-S cell cycle progression. The SWATH comparative proteomic analysis of MDA-MB-231 cells treated with TcPaSK peptide against non-treated cells demonstrated that sublethal concentrations of TcPaSK altered the levels of proteins involved in cell growth and tumor progression. Collectively, our results support a dual role for TcPaSK peptide as an antimicrobial and antiproliferative agent, revealing its versatile therapeutic potential in a wide range of concentrations. Additional studies are needed to explore the effectiveness of TcPaSK in other microorganisms and tumor cell lines to ascertain future prospects and limits of this novel peptide. 

## 6. Patents

Patent application PCT/ES2018070824 titled “Peptide and pharmaceutical compositions of the same for use as an antimicrobial and in cancer treatment”.

## Figures and Tables

**Table 1:** Theoretical predictions of the physicochemical parameters of TcPaSK and Tcdef3 peptides. Molecular mass (MW), net charge at pH = 7 (NC) and isoelectric point (pI) values were calculated from * https://www.bachem.com/service-support/peptide-calculator/. APD defined total hydrophobic ratio (HR), hydrophobicity (H) and Grand Average hydropathy (GRAVY) values were estimated from ** APD3: Antimicrobial Peptide Calculator and Predictor (http://aps.unmc.edu/AP/prediction/prediction_main.php), as well as the theoretical predictions.

	TcPaSK	Tcdef3
**Sequence**	**KVNHAACAAHCLLKRKRGGYCNKRRICVCRN** **31 residues:** *Hydrophobic residues are in red, P and G are in blue, positively charged are in magenta*	**VNHAACAAHCLLKRKRGGYCNKRRICVCR** **29 residues:** *Hydrophobic residues are in red, P and G are in blue, positively charged are in magenta*
MW (daltons) *	3543.30	3301.02
NC *	+9	+8
pI *	10.6	10.5
HR **	45%	48%
H **	0.225	0.296
GRAVY **	−0.545	−0.328
APD Theoretical predictions **	- Peptide may form α-helices and may have at least 6 residues on the same hydrophobic surface. - Peptide may interact with membranes and has the potential to be an antimicrobial peptide.	- Peptide may form α-helices and may have at least 5 residues on the same hydrophobic surface. - Peptide may interact with membranes and has the potential to be an antimicrobial peptide.

**Table 2:** Differentially expressed proteins identified by SWATHS analysis in MDA-MB-231 TNBC cells treated (T) or not (C) with TcPaSK peptide. Shown in bold are proteins exhibiting ≥1.5-fold change T/C.

Peak Name	Group	*t*-Value	*p*-Value	Fold Change C/T
**sp|O43681|ASNA_HUMAN**	**ATPase ASNA1**	**3.92852**	**0.02483**	**4.81**
**sp|P06132|DCUP_HUMAN**	**Uroporphyrinogen decarboxylase**	**4.99399**	**0.00485**	**4.66**
**sp|Q9H2U1|DHX36_HUMAN**	**ATP-dependent RNA helicase DHX36**	**2.90488**	**0.02776**	**3.69**
**sp|Q6PKG0|LARP1_HUMAN**	**La-related protein 1**	**3.78106**	**0.00944**	**3.63**
**sp|Q9NR46|SHLB2_HUMAN**	**Endophilin-B2**	**4.20419**	**0.00903**	**3.44**
**sp|O43583|DENR_HUMAN**	**Density-regulated protein**	**2.88173**	**0.02873**	**3.22**
**sp|P26368|U2AF2_HUMAN**	**Splicing factor U2AF 65 kDa subunit**	**2.82794**	**0.03412**	**3.20**
**sp|Q9UIG0|BAZ1B_HUMAN**	**Tyrosine-protein kinase BAZ1B**	**4.12155**	**0.00622**	**3.19**
**sp|Q92544|TM9S4_HUMAN**	**Transmembrane 9 superfamily member 4**	**4.36180**	**0.01640**	**2.72**
**sp|P28340|DPOD1_HUMAN**	**DNA polymerase delta catalytic subunit**	**2.99222**	**0.04858**	**2.01**
**sp|P54819|KAD2_HUMAN**	**Adenylate kinase 2, mitochondrial**	**2.88071**	**0.02808**	**1.98**
**sp|P46777|RL5_HUMAN**	**60S ribosomal protein L5**	**2.54086**	**0.04416**	**1.66**
**sp|Q99961|SH3G1_HUMAN**	**Endophilin-A2**	**3.82975**	**0.01530**	**1.58**
**sp|P55145|MANF_HUMAN**	**Mesencephalic astrocyte-derived neurotrophic factor**	**2.87810**	**0.04330**	**1.58**
sp|Q05682|CALD1_HUMAN	Caldesmon	2.76783	0.03276	1.40
sp|P67936|TPM4_HUMAN	Tropomyosin alpha-4 chain	2.82893	0.03159	1.36
sp|P37802|TAGL2_HUMAN	Transgelin-2	3.76778	0.00985	1.34
sp|P51114|FXR1_HUMAN	Fragile X mental retardation syndrome-related protein 1	2.57835	0.04190	1.31
sp|P62277|RS13_HUMAN	40S ribosomal protein S13	2.98365	0.02454	1.22
sp|P17812|PYRG1_HUMAN	CTP synthase 1	3.22684	0.03034	1.22
sp|Q9H3H3|CK068_HUMAN	UPF0696 protein C11orf68	5.03897	0.00243	1.20
sp|P55884|EIF3B_HUMAN	Eukaryotic translation initiation factor 3 subunit B	2.84937	0.03000	1.19
sp|Q92841|DDX17_HUMAN	Probable ATP-dependent RNA helicase DDX17	3.09782	0.04655	1.19
sp|Q01105|SET_HUMAN	Protein SET	2.81192	0.04233	1.11
sp|Q9H5V8|CDCP1_HUMAN	CUB domain-containing protein 1	−3.09560	0.03259	0.82
sp|Q9Y6N5|SQRD_HUMAN	Sulfide:quinone oxidoreductase, mitochondrial	−2.72138	0.03583	0.77
sp|O00560|SDCB1_HUMAN	Syntenin-1	−2.95845	0.03792	0.70
sp|P15153|RAC2_HUMAN	Ras-related C3 botulinum toxin substrate 2	−3.38378	0.01906	0.69
**sp|Q16881|TRXR1_HUMAN**	**Thioredoxin reductase 1, cytoplasmic**	**−2.83327**	**0.03590**	**0.63**
**sp|P50281|MMP14_HUMAN**	**Matrix metalloproteinase-14**	**−3.22740**	**0.03678**	**0.52**
**sp|P07203|GPX1_HUMAN**	**Glutathione peroxidase 1**	**−5.48171**	**0.00695**	**0.17**

## Data Availability

No new data were created or analyzed in this study. Data sharing is not applicable to this article.

## Supplementary Materials

The following are available online at https://www.mdpi.com/2076-2607/9/2/222/s1, Figure S1: Quality assessment of TcPaSK peptide structure prediction and topology diagram of TcPaSK peptide derived from the model-template based structural alignment of the four top hits in the PDB data banktitle. Figure S2: Helical wheel projection of the predicted α-helix region of TcPaSK peptide. Table S1: Minimum inhibitory concentration (MIC) of TcPaSK against *S. aureus*. Table S2: Hemolytic activity of TcPaSK peptide against human red blood cells at different concentrations.
