# Supplementary material for: Dual Antimicrobial and Antiproliferative Activity of TcPaSK Peptide Derived from a Tribolium castaneum Insect Defensin
Source: Microorganisms. 2021 Jan 22;9(2):222. doi: 10.3390/microorganisms9020222 (PMC7912591; doi:10.3390/microorganisms9020222)
Supplement: Supplementary file 1 [file microorganisms-09-00222-s001.pdf]

Table S1. Minimum inhibitory concentration (MIC) of TcPaSK against *S. aureus*. Experiments were performed in triplicate.

**TcPaSK concentration (μg/mL)**	**128**	**64**	**32**	**16**	**8**	**4**	**2**	**1**	**0.5**	**0.25**
	-	-	-	+	+	+	+	+	+	+

(+) visible growth appearance.

(-) no visible growth appearance.

Table S2. Hemolytic activity of TcPaSK peptide against human red blood cells at different concentrations. Percentage of haemolysis were calculated as described in Material and Methods using the control hemolytic peptide Melittin. Values are mean ± SE of three independent experiments.

**Concentration (μM)**	**2.35**	**4.7**	**9.4**	**18.75**	**37.5**	**75**
TcPaSK (% hemolysis)	7.28 ± 0.1	10.00 ± 0.7	11.44 ± 2.0	12.7 ± 0.9	14.0 ± 2.4	16.0 ± 3.4

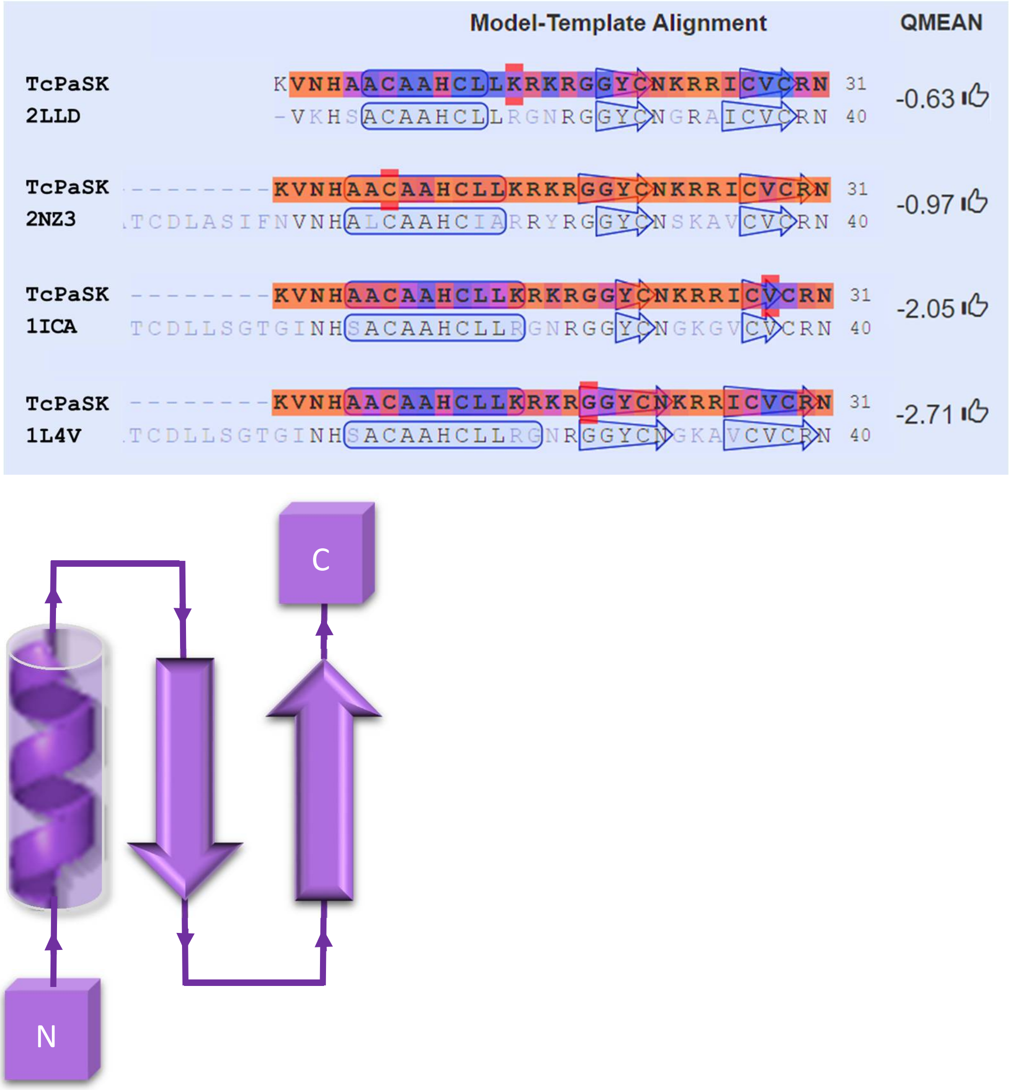

Figure S1. (A) Quality assessment of TcPaSK peptide structure prediction based on the composite scoring function QMEAN (Swiss Model platform https://swissmodel.expasy.org/qmean/) which estimates the likelihood that a given model is of comparable quality to experimental structures [30]. **(B)** Topology diagram of TcPaSK peptide derived from the model-template based structural alignment of the four top hits in the PDB data bank. Diagram shows how the alpha-helix (cylinder with a spiral ribbon inside) is located relative to the beta strands (represented by the large arrows) that forms and antiparallel beta-sheet. Small arrows indicate the directionality of the protein chain, from the N- to the C-terminus.

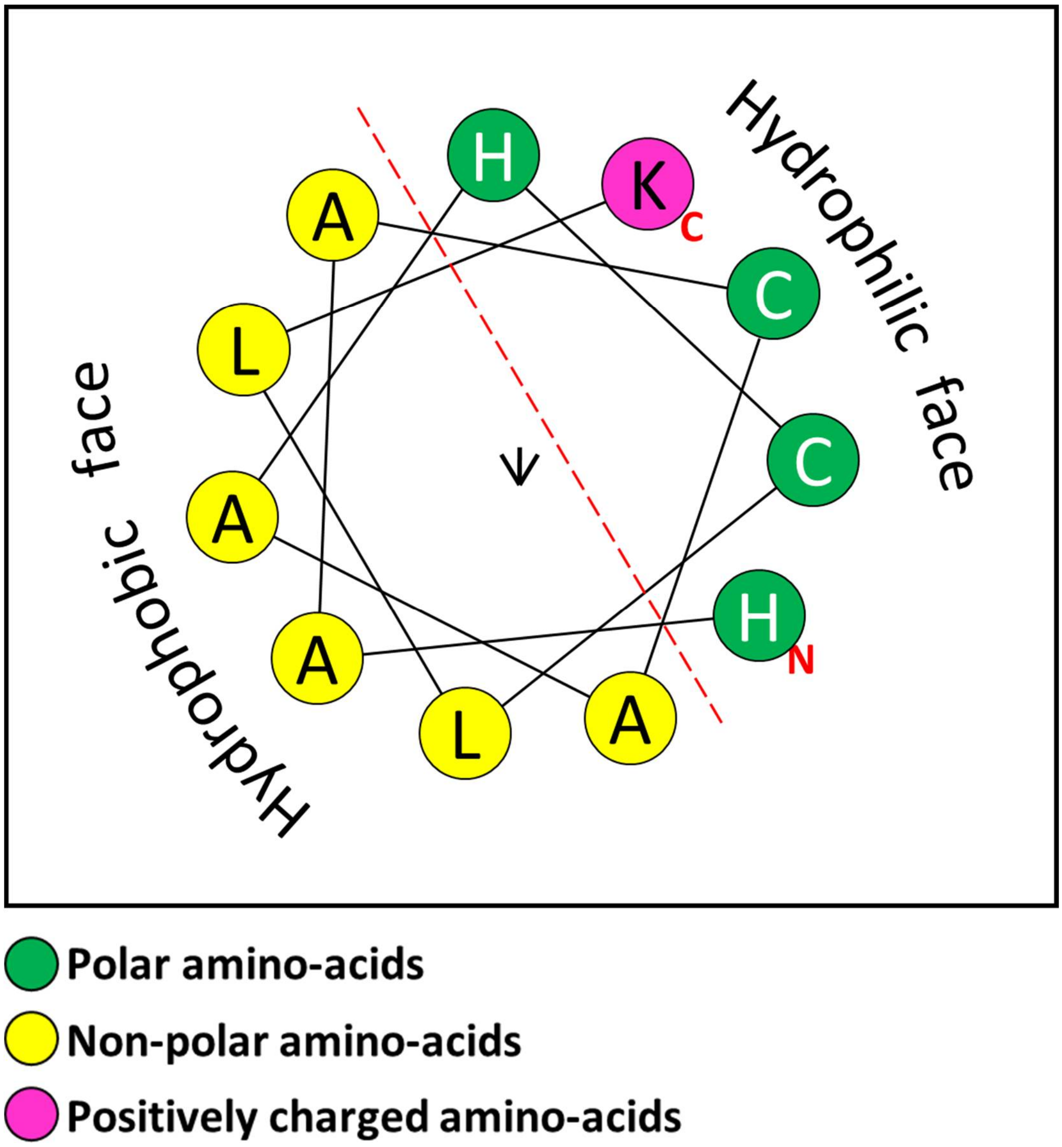

Figure S2. Helical wheel projection of the predicted α-helix region of TcPaSK peptide. Amino acids in yellow color are hydrophobic (nonpolar), amino acids in green color are polar (hydrophilic) and aminoacids in magenta color are positively charged. Red line denotes the spatial separation of the hydrophobic and hydrophilic faces of the amphypatic α-helix. The letters N and C in the projections indicate the N- and C-terminals, respectively. Arrow represents the hydrophobic moment and indicate the direction of summed vectors of hydrophobicity estimated using HELIQUEST software (https://heliquest.ipmc.cnrs.fr/).
